# Effects of intranasal TNFα on granulocyte recruitment and activity in healthy subjects and patients with allergic rhinitis

**DOI:** 10.1186/1465-9921-9-15

**Published:** 2008-01-30

**Authors:** Henrik Widegren, Jonas Erjefält, Magnus Korsgren, Morgan Andersson, Lennart Greiff

**Affiliations:** 1 Department of Otorhinolaryngology, Head & Neck Surgery, Lund University Hospital, Lund, Sweden; 2 Department of Experimental Medical Sciences, Lund University, Lund, Sweden; 3 Department of Clinical Pharmacology, Lund University Hospital, Lund, Sweden

## Abstract

**Background:**

TNFα may contribute to the pathophysiology of airway inflammation. For example, we have recently shown that nasal administration of TNFα produces late phase co-appearance of granulocyte and plasma exudation markers on the mucosal surface. The objective of the present study was to examine indices of granulocyte presence and activity in response to intranasal TNFα challenge.

**Methods:**

Healthy subjects and patients with allergic rhinitis (examined out of season) were subjected to nasal challenge with TNFα (10 μg) in a sham-controlled and crossover design. Nasal lavages were carried out prior to and 24 hours post challenge. Nasal biopsies were obtained post challenge. Nasal lavage fluid levels of myeloperoxidase (MPO) and eosinophil cationic protein (ECP) were analyzed as indices of neutrophil and eosinophil activity. Moreover, IL-8 and α_2_-macroglobulin were analyzed as markers of pro-inflammatory cytokine production and plasma exudation. Nasal biopsy numbers of neutrophils and eosinophils were monitored.

**Results:**

Nasal lavage fluid levels of MPO recorded 24 hours post TNFα challenge were increased in healthy subjects (p = 0.0081) and in patients with allergic rhinitis (p = 0.0081) (*c.f*. sham challenge). Similarly, α_2_-macroglobulin was increased in healthy subjects (p = 0.014) and in patients with allergic rhinitis (p = 0.0034). Lavage fluid levels of ECP and IL-8 were not affected by TNFα challenge. TNFα increased the numbers of subepithelial neutrophils (p = 0.0021), but not the numbers of eosinophils.

**Conclusion:**

TNFα produces a nasal inflammatory response in humans that is characterised by late phase (i.e., 24 hours post challenge) neutrophil activity and plasma exudation.

## Background

Tumour necrosis factor-α (TNFα) is an important pro-inflammatory cytokine of the immune system. Dysregulated TNFα responses are implicated in several inflammatory diseases including rheumatoid arthritis. In this condition, TNFα antagonism, either with recombinant soluble receptors or neutralising antibodies, improves disease activity scores [1].

In human bronchial airways, TNFα is produced by several cell types including macrophages [2], eosinophils [3,4], epithelial cells [5,6], and mast cells [7,8]. Inhalation of TNFα produces a response that is characterized by increased sputum numbers of granulocytes and increased airway responsiveness [9,10]. Furthermore, in refractory asthma, treatment with etanercept, an anti-TNFα measure, is associated with improvement in asthma symptoms, lung function, and airway hyperresponsiveness [11,12]. While these observations suggest an association between TNFα and bronchial airway inflammation, little is known about TNFα-induced nasal airway responses in man.

Focusing on human nasal airways, patients suffering from allergic rhinitis have been shown to feature increased tissue expression of TNFα mRNA and increased nasal mucosal output of TNFα [13-15]. Furthermore, in a preliminary study, we have shown that while nasal challenge with TNFα fails to produce acute effects, it produces late-phase plasma exudation (i.e., extravasation and luminal entry of plasma) [16], a response characteristic of on-going inflammation [17]. We have also observed that this response may be associated with increased nasal mucosal output of granulocyte mediators [16]. While our observations suggest a role for TNFα in upper airway conditions characterised by inflammation, information on the nature of this response is scarce. Further information in this field is warranted for several reasons: it may be used to determine whether or not anti-TNF measures should be evaluated for upper respiratory tract conditions. Furthermore, studies in allergic rhinitis may elucidate specific roles for TNFα in type-1 allergic inflammation, which may not be done in allergic asthma where type-1 features may be blurred by chronic inflammatory features.

The aim of the present study was to examine characteristics of the inflammatory process evoked by human nasal administration of TNFα. Nasal challenges with TNFα were carried out and the response was monitored by nasal lavages and biopsies and by analysis of granulocyte activation and plasma exudation markers as well as tissue numbers of granulocytes. Based on our previous study [16], we focused the evaluation on an observation point 24 hours post challenge. Healthy subjects and patients with allergic rhinitis (examined out of season) were included in order to get a preliminary estimation regarding whether or not these group of subjects differ with regard to TNFα-induced effects.

## Methods

### Study Design

The present study involved nasal administration of a single dose of TNFα (10 μg) in a single blind, sham-controlled (isotonic saline), randomized, and crossover design with a two-week washout period. Nasal lavages were carried out prior to and 24 hours post challenge. Furthermore, nasal mucosal biopsies were obtained 24 hours post-challenge. Lavage fluid levels of myeloperoxidase (MPO), eosinophil cationic protein (ECP), α_2_-macroglobulin, and IL-8 were measured as indices of neutrophil activity, eosinophil activity, plasma exudation, and pro-inflammatory cytokine production, respectively. Numbers of neutrophils and eosinophils in the biopsy material were determined. Nasal symptoms were scored at strategic time-points (below).

### Subjects

Thirteen healthy subjects (5 females, 8 males, aged 20–28 years) and 14 patients with seasonal allergic rhinitis (8 females, 6 males, aged 23–45 years) participated in the study. The protocol was approved by the Regional Ethics Committee and written informed consent was obtained. The study was carried out in compliance with the Helsinki Declaration. For healthy subjects, inclusion criteria were: negative skin prick-test, normal nasal examination, and use of contraceptives (for females). Exclusion criteria were: history of respiratory tract infection within seven days of the start of the study, allergic and non-allergic rhinitis, other nasal disease (structural abnormalities, rhinosinusitis, and polyposis), use of decongestants within seven days of the start of the study, and use of other treatments (except occasional analgesics) within one month of the start of the study. For patients with allergic rhinitis, inclusion and exclusion criteria were the same as described above except that these subjects presented a history of at least two years of seasonal allergic rhinitis and a positive skin-prick test to relevant allergens (patients with positive tests for perennial allergens were excluded). Accordingly, eleven of the allergic subjects were skin-prick positive for both birch and timothy grass allergen, whereas one was sensitized to birch alone and one to timothy grass alone. None of the individuals had positive tests for house dust mite. Eight subjects had positive skin-prick tests for either cat or dog dander, but none of them were exposed to these animals on a regular basis.

### TNFα challenge

Recombinant human TNFα (210-TA/CF, R&D Systems, Abingdon, UK) was diluted with isotonic saline to a 100 μg/ml concentration. The solution was prepared less than one hour before the challenge and it was administered using a spray-device delivering 100 μl per actuation. The dose of 10 μg of TNFα was based on experience from our previous study [16]. Isotonic saline was used for the sham challenge. The challenges were given in a randomized order. The right hand side nasal cavity was challenged at the first visit and the left hand side at the second visit. The reason for using both nasal cavities was to avoid any influence of the first biopsy on the second challenge and sampling procedure. A washout period of two weeks was instituted between the challenges to make the repeated biopsy procedure more tolerable.

### Symptom scores

Nasal symptoms were scored by the subjects prior to TNFα challenge and 24 hours thereafter. Sneezes, secretion, and blockage were each scored on a four-graded scale: 0 for no symptoms, 1 for mild symptoms, 2 for moderate symptoms, and 3 for severe symptoms. The scores were added to a total nasal symptom score (range 0–9).

### Nasal lavages

Nasal lavages with isotonic saline were carried out using a pool-device [18]. The volume of the pool-fluid was 15 ml and it was kept in the nasal cavity for 5 min at each occasion. The lavages were performed immediately after the symptom scoring, before the challenge and before the nasal biopsy at 24 hours. The recovered fluid was centrifuged and the supernatant was homogenized, prepared in aliquots, and frozen (-30°C) for later analysis. The results were expressed as weight of analyte per volume without correction for any dilution of the lavage fluid: correction for dilution may be of little importance when high volume lavages are employed.

### Nasal biopsies

The biopsy was obtained immediately after the second nasal lavage. Topical anaesthesia was applied using a solution of lidocaine hydrochloride (34.0 mg/ml) and nafazoline (0.17 mg/ml), delivered first by a spray-device and thereafter by a cotton swab. The biopsies were taken from the inferior aspect of the inferior turbinate about 0.5 cm from its anterior margin using a cutting-punch forceps with a drilled out punch (2 mm in diameter). Haemostasis was achieved using diathermy.

### Analysis

α_2_-Macroglobulin was measured using a RIA sensitive to 7.8 ng/ml. Rabbit anti-human α_2_-macroglobulin (Dako, Copenhagen, Denmark) was used as anti-serum and human serum (Behringwerke, Marburg, Germany) as standard. Human α_2_-macroglobulin (Capell-Organon, Turnhout, Belgium) was iodinated. Tracer and standard or sample was mixed with antiserum before adding goat anti-rabbit anti-serum (AstraZeneca, Lund, Sweden). The bound fraction was measured using a gamma counter. The intra- and inter-assay coefficients of variation are between 3.8–6.0% and 3.1–7.2%, respectively. IL-8 was measured using a commercially available enzyme-linked immunosorbent assay (R&D Systems, Abingdon, UK). ECP and MPO were also measured using commercially available assays (Phadia, Uppsala, Sweden and Calbiochem, Darmstadt, Germany, repectively). The limits of detection for IL-8, ECP and MPO were 31.2 pg/ml, 2.0 and 1.6 ng/ml, respectively.

The biopsies were immersed overnight in buffered 4% paraformaldehyde (pH 7.2), dehydrated in increasing concentrations of ethanol, embedded in paraffin, and sliced in 3 μm sections. After rinsing in TRIS-buffered saline, the sections were incubated with primary monoclonal antibodies overnight at 4°C. The monoclonal antibody EG2 (Phadia, dilution 1:200), which detects the eosinophil granule protein ECP, was used to identify eosinophils. Neutrophils were detected by a monoclonal antibody against the neutrophil granule protein MPO (Dako, A390, dilution 1:8000). After incubation with primary antibodies specimens were rinsed in TBS buffer and incubated with a secondary antibody (rabbit anti-mouse, Dako Z0109, dilution 1:80) for 40 min at room temperature. After washing in TBS the sections were incubated in mouse APAAP (Dako D0651, dilution 1:50 in 20% NHS) for 30 min, rinsed, and developed using New Fuchsin (Dako) as substrate. Endogenous alkaline phosphatase activity was inhibited by Levamisol (Dako). All sections were counter-stained with Harris's haematoxylin, coated with Aqua Perm mountant (484975 Life Sci. International), dried overnight, and mounted in Kaiser's glycerol gelatin (Merck, Darmstadt, Germany). The sections were blinded and the numbers of ECP-positive (eosinophils) and MPO-positive (neutrophils) subepithelial cells were counted down to a depth of 210 μm below the epithelial basement membrane. The numbers of eosinophils and neutrophils were expressed as cells/mm^2 ^of tissue compartment.

### Statistics

Differences in nasal lavage fluid levels of MPO, ECP, IL-8, and α_2_-macroglobulin as well as biopsy numbers of neutrophils and eosinophils, between observations following TNFα and sham challenge, were analyzed using the Wilcoxon signed rank test. A p-value less than 0.05 was considered significant. Data is presented as mean ± S.E.M.

## Results

One patient with allergic rhinitis was excluded from the study because of airway infection. Otherwise, all individuals complied with the protocol. Nasal administration of TNFα did not produce any nasal symptoms: score 0.8 ± 0.3 post sham challenge and score 0.8 ± 0.2 post TNFα (range 0–9).

**Figure 1:**
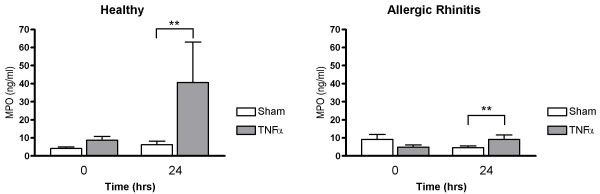
Nasal lavage fluid levels of MPO prior to and 24 hours post TNFα and sham challenge. (**p < 0.01).

**Figure 2:**
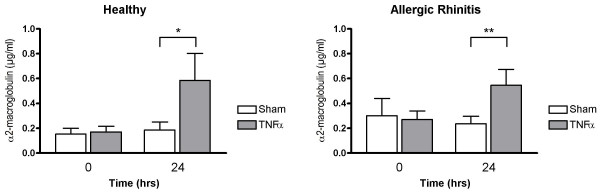
Nasal lavage fluid levels of α_2_-macroglobulin prior to and 24 hours post TNFα and sham challenge. (*p < 0.05, **p < 0.01).

**Figure 3:**
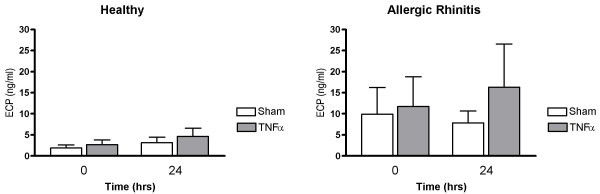
Nasal lavage fluid levels of ECP prior to and 24 hours post TNFα and sham challenge.

**Figure 4:**
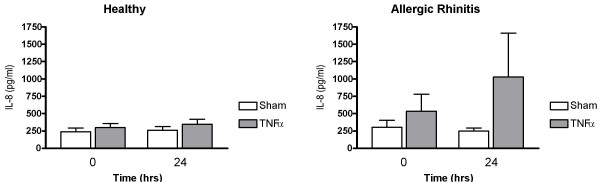
Nasal lavage fluid levels of IL-8 prior to and 24 hours post TNFα and sham challenge.

Nasal lavage fluid levels of MPO recorded 24 hrs post TNFα challenge were increased in healthy subjects (p = 0.0081) and in patients with allergic rhinitis (p = 0.0081) (*c.f*. sham challenge) (Fig. 1). Furthermore, α_2_-macroglobulin was increased in both these study groups (p = 0.014 and p = 0.0034, respectively) (Fig. 2), indicating plasma exudation. ECP and IL-8 were not affected by TNFα challenge (Figs. 3 and 4).

**Figure 5:**
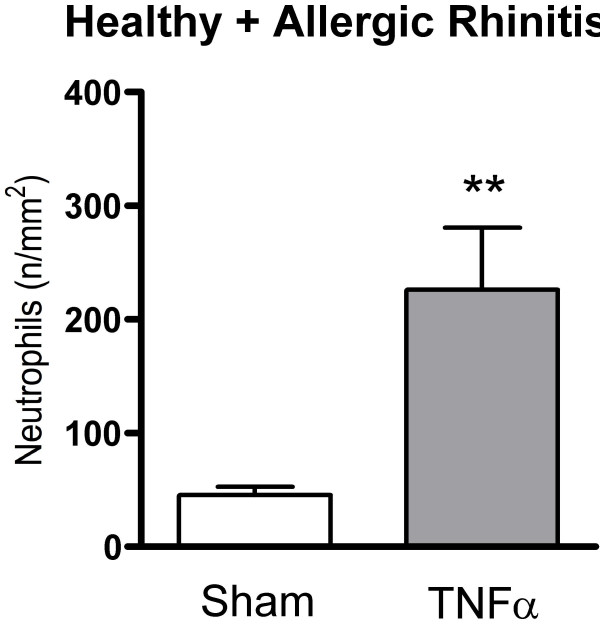
Numbers of subepithelial neutrophils in nasal biopsies obtained 24 hours post TNFα and sham challenge in the study material as a whole. (**p < 0.01).

**Figure 6:**
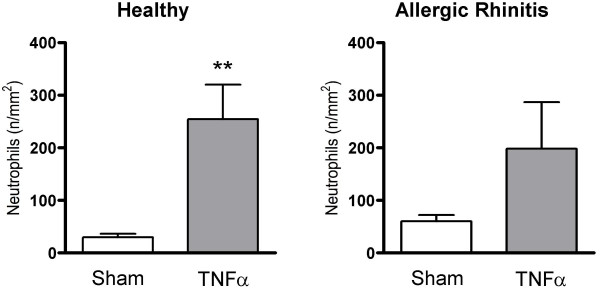
Numbers of subepithelial neutrophils in nasal biopsies obtained 24 hours post TNFα and sham challenge in healthy subjects and patients with allergic rhinitis. (**p < 0.01).

**Figure 7:**
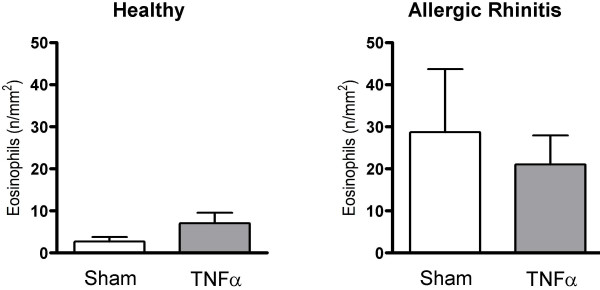
Numbers of subepithelial eosinophils in nasal biopsies obtained 24 hours post TNFα and sham challenge in healthy subjects and patients with allergic rhinitis.

**Figure 8:**
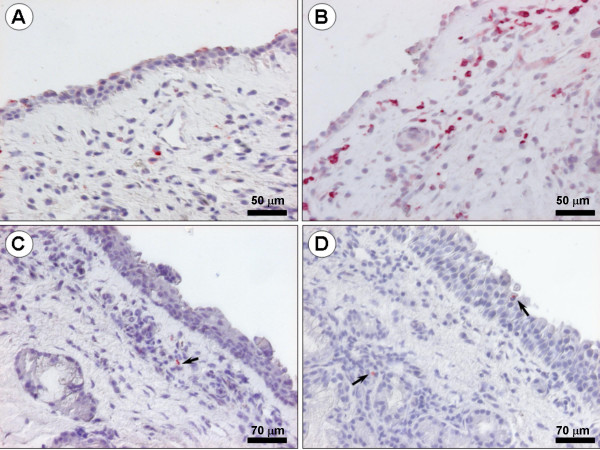
Micrographs showing nasal mucosal neutrophils and eosinophils 24 hours post TNFα and sham challenge. A: Sham-challenged healthy subject: biopsy stained for the neutrophil granule protein MPO. B: TNFα challenged patient with allergic rhinitis: biopsy stained for MPO. C: Sham-challenged healthy subject: biopsy stained for the eosinophil granule protein ECP. D: TNFα challenged healthy subject: biopsy stained for ECP. Nasal biopsy numbers of neutrophils were increased 24 hours after TNFα challenge.

TNFα increased the numbers of subepithelial neutrophils in the study material as a whole (p = 0.0021) (Fig. 5): a subgroup analysis revealed that this change reflected a statistically significant increase in the healthy group (p = 0.0015), but not in the allergic rhinitis group (p = 0.204) (Fig. 6). Nasal biopsy numbers of eosinophils were unaffected by the TNFα challenge (Fig. 7). (Note, a non-significant difference 24 hours post sham challenge between healthy subjects and patients with allergic rhinitis also for eosinophils (p = 0.06, Mann Whitney).) Fig. 8 shows micrographs of nasal mucosal neutrophils and eosinophils 24 hours post TNFα and sham challenge, respectively.

## Discussion

The present study, involving healthy subjects and patients with allergic rhinitis, demonstrates that intranasal TNFα produces a local inflammatory response. Our observation, together with our previous findings [16], suggests that this effect predominantly involves recruitment and activation of neutrophils and a low-grade exudative response 24 hours post challenge. The observation is of interest in terms of nasal airway defence/physiology and in terms of whether or not TNFα should be viewed as a pro-inflammatory factor in respiratory conditions characterised by inflammation.

The dose of TNFα and the time-point for monitoring of the present response was chosen based on our previous observation: the 10 μg dose may be low, but has been shown to produce inflammatory effects 24 hours post challenge [16]. In the present study, the plasma exudation producing effect of TNFα (10 μg) at this time-point was confirmed. Taken together, our observations suggest that TNFα may not *per se *produce acute plasma exudation in human nasal airways (like, e.g., histamine would), but can induce an inflammatory condition that is characterised by "late-phase" plasma exudation. Plasma exudation is a specific feature of inflammation and increased levels of plasma proteins in airway mucosal surface liquids characterize allergic rhinitis and upper respiratory tract infections [19,20]. Accordingly, and supported by observations on increased levels of TNFα in allergic rhinitis [13,14] as well as common cold [21,22], TNFα may be a pro-inflammatory factor in inflammatory conditions of the nasal airway.

Nasal lavage fluid levels of MPO were increased 24 hours post TNFα administration in healthy subjects and in patients with allergic rhinitis (this study). The finding is in agreement with our previous observation in allergic rhinitis, although that particular increase in MPO only reached borderline statistical significance [16]. Moreover, in the present study, nasal biopsy numbers of neutrophils, in the study group as a whole, were significantly increased post TNFα challenge further indicating a neutrophil-active effect of this cytokine. Our finding is in agreement with observations by Thomas *et al*. [9,10], who reported sputum neutrophilia in healthy subjects as well as in patients with mild asthma following TNFα inhalation. Also in agreement, observations *in vitro *have identified TNFα as a factor that attracts [23] and, potentially, activates [24] neutrophil granulocytes. Taken together, available information now indicates that TNFα can be involved in neutrophil recruitment and activity in human airways. We have no specific explanation for the present numerically higher levels of MPO observed in healthy subjects (*c.f*. patients with allergic rhinitis) following TNFα challenge. However, this difference is not statistically significant (p = 0.5, Mann Whitney).

In our previous study, involving patients with allergic rhinitis examined out of season, we observed moderately increased lavage fluid levels of ECP 24 hours after nasal TNFα challenge [16]. In contrast, in the present study, this observation could not be confirmed. We have no specific explanation for the discrepant findings, but a variable eosinophil activity at baseline may be one option: Thomas *et al*. observed that inhalation of TNFα to mild asthmatics increased eosinophil infiltration [9] whereas this was not the case in healthy subjects [10]. In the present study, the biopsy analysis, which provides an additional aspect of cellular activity, showed that TNFα did not produce recruitment of eosinophils. Accordingly, our overall conclusion is that TNFα administered to patients with allergic rhinitis out of season, when there may be some degree of nasal eosinophilia but not at all as marked as during season allergen exposure, does not produce any marked pro-eosinophil effects. Further studies are warranted to examine effects of TNFα on eosinophil activities, tentatively involving patients with on-going allergic rhinitis.

The present study was not powered to discriminate between healthy subjects and patients with allergic rhinitis, but may allow for a preliminary comparison. The only difference in TNFα induced effects between the groups was that the nasal mucosal numbers of neutrophils were increased in healthy subjects following TNFα challenge but not in patients with allergic rhinitis. However, TNFα did produce a numerical increase in neutrophils also in allergic rhinitis, which did not reach statistical significance (possibly due to a somewhat higher baseline). Furthermore, MPO, as marker of neutrophil activity, was significantly increased in both study groups. Taken together, no marked differences emerged between healthy subjects and patients with allergic rhinitis regarding TNFα-induced effects. However, further studies may be warranted in order to elucidate whether TNFα has a specific role in allergic airway conditions: such studies are warranted based on observations that patients with allergic rhinitis feature increased tissue expression of TNFα mRNA and TNFα protein [13-15]. Moreover, a basis for further exploration may be the recent observation that mice sensitized to ovalbumin and subjected to gene therapy (soluble TNFα receptor IgGFc fusion gene) present decreased symptoms and attenuated aggregation of mast cells, eosinophils, and IL-5 positive cells following nasal ovalbumin challenge [25]. Tentatively, further human *in vivo *studies may involve intervention with anti-TNFα measures in allergic rhinitis at seasonal allergen exposure.

Anti-TNFα drugs, including etanercept, have emerged as a possible treatment option for refractory asthma [11,12]: reduction of symptoms, improvement in lung function, and reduction of bronchial hyperresponsiveness as a result of anti-TNFα intervention. A similar use of anti-TNFα drugs in allergic rhinitis is in our opinion not likely, in part reflecting that existing treatment options are very good and in part inherited difficulties with current anti-TNFα approaches. However, the human nasal airway and allergic rhinitis can be used to monitor effects of TNFα (this study) and anti-TNFα drugs. This may be particularly valid since there are key similarities between inflammatory processes of the upper and lower airways, since observations made in upper airway conditions frequently can be translated to the bronchial airway diseases, and since nasal airway studies are easier to perform. In this context, the present findings suggest, indirectly, that beneficial effects of anti-TNFα drugs in refractory asthma may reflect anti-neutrophil rather than anti-eosinophil effects.

## Conclusion

Topical TNFα produces a nasal inflammatory response in humans that potentially is mediated through increased neutrophil activity.

## Competing interests

The author(s) declare that they have no competing interests.

## Authors' contributions

All authors participated in the design process of the study. HW, MK, MA, and LG carried out the challenge, lavage/biopsy, and lavage analysis procedures. HW and JE performed the immunohistochemistry. HW and LG drafted the manuscript. All authors read and approved the final manuscript.
